# The diagnostic accuracy of nanopore sequencing in Tuberculous Lymphadenitis: Systematic review and meta-analysis protocol

**DOI:** 10.1371/journal.pone.0325370

**Published:** 2025-06-10

**Authors:** Liwei Yao, Caifang Bu, Lijun Xiang

**Affiliations:** Department of Nursing, Hangzhou Red Cross Hospital, Hangzhou, Zhejiang, China; Stellenbosch University, SOUTH AFRICA

## Abstract

**Background:**

Tuberculous lymphadenitis (TL) is a common case of extrapulmonary tuberculosis caused by *Mycobacterium tuberculosis* (MTB). The current use of nanopore sequencing in TL is limited. The aim of our study is to complete a systematic review and meta-analysis protocol to assess the performance of nanopore sequencing in TL.

**Methods:**

English databases (including Medline via PubMed, Cochrane Library, and Embase) and Chinese databases (including Wanfang Database and China National Knowledge Infrastructure) will be searched for literature related to the research topic. We designed eligibility criteria based on PICT (Population, Index Test, Comparator Test and Target Condition). We will extract relevant data from the included articles when they have been identified. The quality of the studies will be assessed using the Quality Assessment of Diagnostic Accuracy Studies (QUADAS-2) tool. We will use *midas* package in STATA for the calculation of the pooled effect values and the corresponding forest plots when the final number of included studies is more than four, and Meta-DiSc for the calculation of the pooled effect values and the forest plots when the number of included studies is less than four. When the results of different reference gold standards are reported simultaneously in the same literature, we will extract the corresponding TP, FP, FN, and TN values according to the different reference gold standards and analyze them independently. *I*^*2*^ statistic will be used to quantify inter-study heterogeneity. If substantial heterogeneity (*I²* > 50%) is detected, potential sources will be investigated through subgroup, meta-regression, and sensitivity analysis.

**Conclusion:**

We conducted this protocol according to the guidelines and submitted it to a peer-reviewed journal to improve our protocol through peer review and ultimately better guide the completion of the systematic review and meta-analysis.

**Systematic review registration:**

PROSPERO Registration number: CRD42024595521

## Introduction

Tuberculous lymphadenitis (TL) is a common form of extrapulmonary tuberculosis (EPTB), accounting for approximately 30–40% of EPTB cases and 5–10% of all tuberculosis cases [1]. It is caused by infection of the lymph nodes with *Mycobacterium tuberculosis* (MTB) [1]. TL can occur in lymph nodes throughout the body such as the cervical, axillary, inguinal, and mediastinal lymph nodes [2]. TL is easily confused with lymphadenitis due to other causes. If proper treatment is not given, TL can lead to abscess formation, skin necrosis, sinus tract formation, prolonged nonhealing of the wound, and even bronchial or esophageal fistulae (when TL occurs in the mediastinum), which affects the patient’s quality of life and prognosis [3]. Obtaining lymph node specimens usually requires invasive procedures such as lymph node biopsy, core needle biopsy (CNB) and fine-needle aspiration (FNA) [4]. How to diagnose TL effectively with minimal trauma and the fewest number of invasive procedures has always been a concern for clinicians.

Traditional classical acid-fast bacilli (AFB) smear and culture are the predominantly applied modalities in TL diagnosis, but they may not be the first modality of choice when specimen volume is limited [5]. The reason is that although AFB smear is simple and easy to use, its sensitivity (32.0%) is still extremely low and cannot effectively detect MTB, and its results are also affected by the microscopist [6]. AFB culture, although it is the reference standard for the diagnosis of tuberculosis, requires several weeks to yield results, which is not conducive to timely diagnosis and precise treatment. On the other hand, in TL, the positivity rate of AFB culture is still on low side (45.4%) [7]. The development of molecular biology techniques has led to dramatic improvements in the rapid diagnosis of tuberculosis [8]. Among the many molecular tests available, Xpert MTB/RIF is one of the most commonly employed modalities [9]. Its use in the timely diagnosis of TL is likewise widespread [10]. A systematic review and meta-analysis showed that the diagnostic sensitivity of Xpert MTB/RIF for TL was 79%−84% comparing different reference gold standards [11]. This suggests that the sensitivity of molecular testing for TL diagnosis still has room for improvement.

Genome sequencing may be able to be a dark horse in the timely diagnosis of tuberculosis [12]. Nanopore sequencing is a third-generation sequencing method, which has the advantages of lightweight testing equipment, long sequencing length, and real-time monitoring compared to next-generation sequencing (NGS) [13]. In the field of tuberculosis, nanopore sequencing has demonstrated significantly better diagnostic performance than Xpert MTB/RIF, providing an effective tool for timely diagnosis of tuberculosis [14,15]. The current use of nanopore sequencing in TL is limited, and there is no systematic review or meta-analysis on the diagnostic performance of nanopore sequencing in TL. Based on this, we intend to conduct a systematic review and meta-analysis to systematically assess the diagnostic performance of nanopore sequencing in TL. The aim of our current study is to conduct the protocol of that systematic review and meta-analysis to guide the implementation of that study.

## Methods

### Design and registration

The study protocol was developed and registered on PROSPERO (CRD42024595521). We carried out the writing of the protocol on the basis of the Preferred reporting items for systematic review and meta-analysis protocols (PRISMA-P) statement [16]. When the systematic review and meta-analysis is completed, we will report the relevant results based on the Preferred Reporting Items for a Systematic Review and Meta-analysis of Diagnostic Test Accuracy Studies: The PRISMA-DTA Statement [17]. Systematic review and meta-analysis does not involve human beings and does not require ethical approval.

### Eligibility criteria

We designed eligibility criteria based on PICT (Population, Index Test, Comparator Test and Target Condition). Population (P): Patients with presumed TL. Index test (I): Nanopore sequencing. Comparator Test (C): MTB culture and final clinical diagnosis. Target condition (T): TL is the target condition. This protocol places no restrictions on the study type, and any study that reports on relevant diagnostic outcomes can be included. MTB culture and final clinical diagnosis will be used as a reference gold standard to evaluate the diagnostic accuracy of nanopore sequencing for TL. Studies that report a clear gold standard and have accessed to the four values of true positive (TP), false positive (FP), false negative (FN), and true negative (TN) will be used as eligible studies for further analysis. Studies that are not directly accessible but could obtain relevant data by contacting the corresponding author could also be included.

### Exclusion criteria

Exclusion criteria: Studies for which TP, FP, FN, TN values are not available by various methods, meta-analysis, reviews, case reports, case series, studies with abstracts only but no full text, studies published in languages other than Chinese and English.

### Information sources

We will use both Chinese and English databases as information sources to search for studies related to the research topic. Chinese databases include Wanfang Database and CNKI (China National Knowledge Infrastructure), and English databases include Medline via PubMed, Cochrane Library, and Embase. We will conduct the first search in December 2025 and an upgraded search prior to data analysis.

### Search strategy

The corresponding search strategy is designed for each database based on the relevant eligibility criteria (Liwei Yao and Caifang Bu). The following shows the search strategy in PubMed.

#1 “Tuberculosis, Lymph Node”[Mesh] OR “Lymphadenitis, Cervical Tuberculous” OR “Tuberculous Lymphadenitis, Cervical” OR “Lymph Node Tuberculoses” OR “Lymph Node Tuberculosis” OR “Tuberculoses, Lymph Node” OR “Lymphadenitis, Tuberculous” OR “Tuberculous Lymphadenitis” OR Scrofula OR Scrofulas OR “Mycobacterial Cervical Lymphadenitis” OR “Cervical Lymphadenitis, Mycobacterial” OR “Lymphadenitis, Mycobacterial Cervical” OR “Cervical Tuberculous Lymphadenitis”
#2 “Extrapulmonary tuberculosis” OR “Extra pulmonary tuberculosis” OR EPTB
#3 #1 OR #2
#4 “Nanopore Sequencing”[Mesh] OR “Nanopore Sequencing*” OR “Sequencing, Nanopore” OR “third generation sequencing” OR “Oxford Nanopore Technolog*” OR ONT
#5 #3 AND #4

### Literature screening and selection

Endnote V9.2 will be used to manage the relevant studies found from the various database searches, and after using Endnote for the exclusion of duplicate literature, two independent reviewers (Liwei Yao and Caifang Bu) will each determine if the literature meets the eligibility criteria established by this protocol by reading the titles, abstracts, then full texts of the literature. The final inclusion of literature will be determined through screening and cross-checked, and areas of discrepancies will be resolved through consensus with a senior reviewer (Lijun Xiang).

### Data extraction

After screening the literature and identifying the final literature to be included, we will extract the necessary data from the literature for the next step of analysis. This task will be given to these two researchers who conducted the literature screening. Extracted data mainly included the year of publication of the literature, the name of the first author, the country where the study was carried out, the type of study, the four values of the diagnostic cross-tabulation (TP, FP, FN,TN), the number of included patients, the type of patients (adult or not), the method of patient screening (consecutive or not), the AIDS status, the type of specimen (tissue or pus or CNB or FNA), lymph node site (cervical or axillary or inguinal or mediastinal), the status of the specimen (fresh or frozen), the specimen processing method, the type of nanopore sequencing (targeted amplification or not), reference gold standard. The two independent researchers will carry out data extraction separately and the treatment of areas of disagreement will be the same as in the literature screening stage.

### Quality evaluation

The quality of the included literature will be assessed using the Quality Assessment of Diagnostic Accuracy Studies (QUADAS-2) tool [18]. We will assess this on an entry-by-entry basis based on the entries and draw a graph of the quality assessment. Two independent reviewers (Liwei Yao and Caifang Bu) will separately evaluate the quality of the included studies, and the place of disagreement will be decided by discussion with a third reviewer (Lijun Xiang).

### Publication bias

Guided by the guidelines of the PRISMA-DTA Statement, the meta-analysis of DTA does not require the evaluation of publication bias because the current method of evaluating publication bias is still flawed for DTA [17].

### Data synthesis and statistical analysis

The pooled sensitivity, specificity, positive predictive value (PPV), negative predictive value (NPV), and area under the curve (AUC) and their 95% confidence intervals using the TP, FP, FN and TN values will be calculated firstly. We will use *midas* package in STATA (v15.0; Stata Corp., College Station, TX, the USA) for the calculation of the pooled effect values and the corresponding forest plots when the final number of included studies is more than four, and Meta-DiSc (v1.4) for the calculation of the pooled effect values and the forest plots when the number of included studies is less than four. When the results of different reference gold standards are reported simultaneously in the same literature, we will extract the corresponding TP, FP, FN, and TN values according to the different reference gold standards and analyze them independently. *I*^2^ statistic will be used to quantify inter-study heterogeneity, with *I*^2^ greater than 50% suggesting substantial inter-study heterogeneity and *I*^2^ less than 50% suggesting that inter-study heterogeneity is insubstantial. If substantial heterogeneity (I² > 50%) is detected, potential sources will be investigated through subgroup, meta-regression, and sensitivity analysis. Subgroup and meta-regression analysis will be analyzed use these following parameters: the type of study, the type of patients, the method of patient screening, the AIDS status, the type of specimen, lymph node site, the status of the specimen, the specimen processing method, the type of nanopore sequencing. Conducting a sensitivity analysis will be done by excluding a study and presenting the analysis results with and without the study to evaluate whether this study is a high-risk study or not. The sensitivity analysis will evaluate the reliability and robustness of the pooled results.

## Discussion

Timely diagnosis and targeted therapeutic interventions are critical to mitigating complications and improving TL outcomes. The accuracy of nanopore sequencing in the timely diagnosis of pulmonary tuberculosis is excellent [19]. It is plausible that nanopore sequencing can also play a great role in TL. We hope to specifically evaluate and analyze the performance of nanopore sequencing in TL through systematic review and meta-analysis. In order to make this systematic review and meta-analysis more in line with the requirements of the relevant guidelines, we firstly formulated this protocol and attempted to submit for publication in a journal with a peer-review process, and then made relevant modifications through the evaluation of the relevant reviewers, so as to make this protocol more perfect.

## Supporting information

S1 File Preferred Reporting Items for Systematic review and Meta-Analysis Protocols (PRISMA-P) checklist. (DOC)

## References

[1] World Health Organization. Global Tuberculosis Report 2024. World Health Organization; 2024.

[2] Shen Y, Fang L, Ye B, Xu X, Yu G, Zhou L. The role of core needle biopsy pathology combined with molecular tests in the diagnosis of lymph node tuberculosis. Infect Drug Resist. 2022;15:335–45. doi: 10.2147/IDR.S350570 35140479 PMC8818765

[3] Yu G, Lin T, Yu Y, Chen P, Chen M, Zhang Y, et al. Application of Mycobacterium tuberculosis RNA for the rapid diagnosis of lymph node tuberculosis using different specimens. Infect Drug Resist. 2023;16:179–87. doi: 10.2147/IDR.S392045 36636372 PMC9831075

[4] Yao L, Xu X, Chen G, Shen Y, Jiang W. Comparison of the accuracy of two different molecular tests for the diagnosis of tuberculous lymphadenitis using core needle biopsy specimens: a diagnostic accuracy study. Int J Gen Med. 2022;15:5237–46. doi: 10.2147/IJGM.S367127 35655654 PMC9153996

[5] Mohapatra PR, Janmeja AK. Tuberculous lymphadenitis. J Assoc Physicians India. 2009;57:585–90. 20209720

[6] Mulugeta F, Tiruneh M, Abebe B, Yitayew G, Ayehubizu Z, Getahun M, et al. Evaluation of modified bleach technique for the detection of acid fast bacilli in lymph node aspirate at the University of Gondar Comprehensive Specialized Hospital, Northwest Ethiopia. J Clin Tuberc Other Mycobact Dis. 2022;28:100328. doi: 10.1016/j.jctube.2022.100328 35990771 PMC9386082

[7] Ghariani A, Jaouadi T, Smaoui S, Mehiri E, Marouane C, Kammoun S, et al. Diagnosis of lymph node tuberculosis using the GeneXpert MTB/RIF in Tunisia. Int J Mycobacteriol. 2015;4(4):270–5. doi: 10.1016/j.ijmyco.2015.05.011 26964807

[8] Dicks KV, Stout JE. Molecular diagnostics for mycobacterium tuberculosis infection. Annu Rev Med. 2019;70:77–90. doi: 10.1146/annurev-med-040717-051502 30125128

[9] Kohli M, Schiller I, Dendukuri N, Yao M, Dheda K, Denkinger CM, et al. Xpert MTB/RIF Ultra and Xpert MTB/RIF assays for extrapulmonary tuberculosis and rifampicin resistance in adults. Cochrane Database Syst Rev. 2021;1(1):CD012768. doi: 10.1002/14651858.CD012768.pub3 33448348 PMC8078545

[10] Meng X, Fu H, Jia W, Wang Y, Yang G. A comparative study of ultrasound-guided puncture biopsy combined with histopathology and Xpert MTB/RIF in the diagnosis of lymph node tuberculosis. Front Public Health. 2023;10:1022470. doi: 10.3389/fpubh.2022.1022470 36703810 PMC9872513

[11] Yu G, Zhong F, Ye B, Xu X, Chen D, Shen Y. Diagnostic accuracy of the xpert MTB/RIF assay for lymph node tuberculosis: a systematic review and meta-analysis. Biomed Res Int. 2019;2019:4878240. doi: 10.1155/2019/4878240 31236407 PMC6545759

[12] Cohen KA, Manson AL, Desjardins CA, Abeel T, Earl AM. Deciphering drug resistance in Mycobacterium tuberculosis using whole-genome sequencing: progress, promise, and challenges. Genome Med. 2019;11(1):45. doi: 10.1186/s13073-019-0660-8 31345251 PMC6657377

[13] Wang Y, Zhao Y, Bollas A, Wang Y, Au KF. Nanopore sequencing technology, bioinformatics and applications. Nat Biotechnol. 2021;39(11):1348–65. doi: 10.1038/s41587-021-01108-x 34750572 PMC8988251

[14] Yu G, Shen Y, Zhong F, Zhou L, Chen G, Fang L, et al. Diagnostic accuracy of nanopore sequencing using respiratory specimens in the diagnosis of pulmonary tuberculosis. Int J Infect Dis. 2022;122:237–43. doi: 10.1016/j.ijid.2022.06.001 35671950

[15] Zhou L, Zou X, Hu Q, Hua H, Qi Q. Determination of the diagnostic accuracy of nanopore sequencing using bronchoalveolar lavage fluid samples from patients with sputum-scarce pulmonary tuberculosis. J Infect Chemother. 2024;30(2):98–103. doi: 10.1016/j.jiac.2023.09.014 37714266

[16] Shamseer L, Moher D, Clarke M, Ghersi D, Liberati A, Petticrew M, et al. Preferred reporting items for systematic review and meta-analysis protocols (PRISMA-P) 2015: elaboration and explanation. BMJ. 2015;350:g7647. doi: 10.1136/bmj.g7647 25555855

[17] McInnes MDF, Moher D, Thombs BD, McGrath TA, Bossuyt PM, and the PRISMA-DTA Group, et al. Preferred reporting items for a systematic review and meta-analysis of diagnostic test accuracy studies: the PRISMA-DTA Statement. JAMA. 2018;319(4):388–96. doi: 10.1001/jama.2017.19163 29362800

[18] Whiting PF, Rutjes AWS, Westwood ME, Mallett S, Deeks JJ, Reitsma JB, et al. QUADAS-2: a revised tool for the quality assessment of diagnostic accuracy studies. Ann Intern Med. 2011;155(8):529–36. doi: 10.7326/0003-4819-155-8-201110180-00009 22007046

[19] Yang J, Ye W, Zhang C, Lin W, Mei L, Liu S, et al. Accuracy of nanopore sequencing as a diagnostic assay for pulmonary tuberculosis versus smear, culture and xpert MTB/RIF: a head-to-head comparison. Trop Med Infect Dis. 2023;8(9):441. doi: 10.3390/tropicalmed8090441 37755902 PMC10535524

